# Assessing quality of life in a clinical study on heart rehabilitation patients: how well do value sets based on given or experienced health states reflect patients’ valuations?

**DOI:** 10.1186/s12955-016-0453-3

**Published:** 2016-03-22

**Authors:** Reiner Leidl, Bernd Schweikert, Harry Hahmann, Juergen M. Steinacker, Peter Reitmeir

**Affiliations:** Institute for Health Economics and Health Care Management, Helmholtz Zentrum München, Postfach 1129, 85758 Neuherberg, Germany; Munich Center of Health Sciences, Ludwig-Maximilians-University, Ludwigstr. 28 RG, 80539 Munich, Germany; Mapi Group, Konrad-Zuse-Platz 11, 81829 Munich, Germany; Waldburg-Zeil Kliniken – Klinik Schwabenland, Waldburgallee 3-5, 88316 Isny-Neutrauchburg, Germany; Division of Sports and Rehabilitation Medicine, Dept. Internal Medicine II, Ulm University Medical Centre, Frauensteige 6, 89075 Ulm, Germany

**Keywords:** EQ-5D-3L, Decision utility, Experience-based valuation, Patient benefit, Patient-reported outcome

## Abstract

**Background:**

Quality of life as an endpoint in a clinical study may be sensitive to the value set used to derive a single score. Focusing on patients’ actual valuations in a clinical study, we compare different value sets for the EQ-5D-3L and assess how well they reproduce patients’ reported results.

**Methods:**

A clinical study comparing inpatient (*n* = 98) and outpatient (*n* = 47) rehabilitation of patients after an acute coronary event is re-analyzed. Value sets include: 1. Given health states and time-trade-off valuation (GHS-TTO) rendering economic utilities; 2. Experienced health states and valuation by visual analog scale (EHS-VAS). Valuations are compared with patient-reported VAS rating. Accuracy is assessed by mean absolute error (MAE) and by Pearson’s correlation ρ. External validity is tested by correlation with established MacNew global scores. Drivers of differences between value sets and VAS are analyzed using repeated measures regression.

**Results:**

EHS-VAS had smaller MAEs and higher ρ in all patients and in the inpatient group, and correlated best with MacNew global score. Quality-adjusted survival was more accurately reflected by EHS-VAS. Younger, better educated patients reported lower VAS at admission than the EHS-based value set.

EHS-based estimates were mostly able to reproduce patient-reported valuation. Economic utility measurement is conceptually different, produced results less strongly related to patients’ reports, and resulted in about 20 % longer quality-adjusted survival.

**Conclusion:**

Decision makers should take into account the impact of choosing value sets on effectiveness results. For transferring the results of heart rehabilitation patients from another country or from another valuation method, the EHS-based value set offers a promising estimation option for those decision makers who prioritize patient-reported valuation. Yet, EHS-based estimates may not fully reflect patient-reported VAS in all situations.

**Electronic supplementary material:**

The online version of this article (doi:10.1186/s12955-016-0453-3) contains supplementary material, which is available to authorized users.

## Background

Quality of life is a key endpoint in a number of clinical studies. Its measurement requires collection of data on the dimensions and items by which quality of life is being described. In order to gain an overall result, an aggregation step is needed that can be performed by a researcher—such as defining the average across all items as the aggregate—or by an individual’s valuation expressing subjective summary assessment.

This paper considers alternative options to aggregate results by the response of individuals. The valuation of health states is known to vary widely between countries [1–3]. Clinical studies may include patients from different countries, which may influence quality of life results by varying valuations. In addition, health care decision makers and regulators may question whether valuation is appropriate if it has been derived from another population, or by another method than they require. In these cases, results may incur methodological biases when quantifying quality of life endpoints. If access to original study data is provided, the impact of the required approach for valuation can be analyzed by re-valuing health states reported by estimates of the respective value from a population study, that is by using a so-called value set.

Approaches to valuing quality of life may be crucial for study results. Methodologically, valuation can differ in various aspects. Patients or the general population may be asked to perform the valuation. For direct valuation, the visual analog scale (VAS) may be used, or a choice-based method such as the time-trade-off method (TTO) [4, 5]. The health states being valued could be the individual’s own, just experienced health state (EHS), or they could be hypothetical, given health states (GHS). When referring to quality-adjusted life years (QALYs) in economic evaluation studies, quality of life measures are most often integrated using a choice-based valuation of GHS. Such utilities reflect the ex-ante preferences of individuals with regard to health. Typically, they are elicited at the population level, thought to reflect population preferences, and thus used to inform decisions on allocating health care resources [6]. This procedure is well established in decision practice: for example, the UK National Institute for Health and Care Excellence (NICE) has been using this type of information for more than 15 years.

Critical voices have also been raised concerning the theoretical foundations of using such community preferences for allocating health care funds [7]. In addition, some jurisdictions require measurement of patient benefit as the primary indicator in order to decide upon health care technologies. In Sweden, the Dental and Pharmaceutical Benefits Agency prefers EHS-based valuation over GHS-based valuation [8]. In Germany, the Social Code, Book V § 35 1(b) defines patient relevant benefit, including quality of life, as the key effect criterion; this does not refer to ex-ante preferences of the population.

In recent years, several EHS-based value sets have been developed that estimate the individual’s valuation of his/her own health state [8–10]. EHS-based value sets have been used in a range of epidemiological and clinical studies, including diabetes [11], stroke [12], hip replacement [13, 14], inflammatory bowel disease [15], and chronic diseases [16]. EHS-based value sets predict how an average person experiencing a health state would value this health state. For decision makers who focus on patient benefit, EHS-based value sets may thus provide a substitutive valuation in situations where context-specific, patient-reported valuation is lacking. As a pre-condition to such substitution in quality of life measurement, the value set has to accurately predict the patient’s valuation as well as the valuation of patient subgroups, such as patients in the treatment arms of a trial.

This paper assumes the perspective of a decision maker who requests evidence on patient relevant benefit and thus would prefer patients to directly value their own health states. If this is not available, GHS-based and EHS-based value sets derived from population studies could provide options for a second-best solution. In order to assess the performance of such second-best choices, the paper uses quality of life data from a published, clinical study of heart rehabilitation patients. As reference for the endpoint of quality of life, patient-reported valuations are taken. The paper starts out from the counterfactual assumption that the latter are lacking and thus uses two value sets for valuation. Indeed, the clinical study has collected patient-reported valuations. The paper then investigates how well the value sets reflect patients’ valuations.

### Methods

The clinical study re-analyzed here compared inpatient and outpatient rehabilitation of patients following an acute cardiac event. The study was conducted in Germany and labeled SARAH (Stationäre versus ambulante Rehabilition nach akutem Herzereignis). Results have been presented elsewhere, showing that, over 3 weeks of intervention and a 12-month follow-up, inpatient and outpatient rehabilitation did not differ significantly with regard to the primary medical endpoint of event-free survival, combining myocardial infarction, stroke, heart failure, life-threatening rhythm events, unstable angina, and death [17], and also did not differ significantly with regard to generic quality of life and cost-effectiveness [18]. The study was carried out according to the Declaration of Helsinki, and was approved by the Institutional Ethics Board of Ulm University. Written informed consent was obtained from all participants. With inpatient rehabilitation representing standard care in this context, feasibility of randomization had to be clarified first. The study thus used a comprehensive cohort design [19]: Patients who had agreed to participate were offered the option of being randomized and, if they refused, were offered the option to choose a treatment arm. Included were patients below 66 years of age, with myocardial infarction occurring less than 3 months before admission to the rehabilitation hospital. Some 163 patients met the inclusion criteria and were recruited. Only four patients agreed to randomization; of the rest, 112 patients starting in the inpatient rehabilitation arm and 51 patients receiving outpatient rehabilitation were allocated on a preference basis. Patient enrolment started in 2002 and study follow-up ended in 2005. For the methodological re-analysis in the present study, all patients with quality of life measurements included in the cost-effectiveness study were used. To compare valuation measurement, we restricted this study to observed measurements, disregarding imputation.

Quality of life was measured using the EQ-5D-3L, a standardized instrument that is available in more than 170 official language versions [20]. A comprehensive review on the use of the EQ-5D-3L in cardiovascular diseases found 60 application studies and ten studies that analyzed validity or reliability, with the results clearly supporting this use. However, results were not stratified with regard to the use of different types of valuation methods [21]. For German heart rehabilitation patients, the EQ-5D-3L has also been shown to be a valid and reliable tool [22]. In the re-analyzed study, patients were requested to fill in the EQ-5D-3L descriptive system and the VAS at six points in time: admission, discharge, and after 3, 6, 9, and 12 months of follow-up (FU).

We tested the use of two national value sets that have been derived from German population studies [9, 23]. They differ with regard to the key methodological characteristics of valuing quality of life mentioned above, including the additional characteristic of scale adjustment (Table 1). The traditional health economic approach based on GHS-TTO uses the valuation of death in order to anchor the lower end of the scale. The EHS-based approach cannot incorporate anchoring for the state of being dead. For comparability, all the results reported in this paper have been transferred to the 0 to 100 scale.

**Table 1:** Approaches studied to value quality of life

Valuation		Patients’ VAS (reference)	GHS-TTO Germany	EHS-VAS Germany
Who values?	Patient	X		
	Population (value set)		X	X
What is being valued?	Experienced health state	X		X
	Hypothetical health state		X	
How is it valued?	Directly (VAS)	X		X
	Choice-based (TTO)		X	
Scale adjustment	None	X		X
	Anchoring for death		X	
Endpoint when multiplied by time	QALYs (utilities)		X	
	Quality-adjusted survival	X		X

Legend: *VAS* visual analog scale, *GHS* given health states, *EHS* experienced health states, *TTO* time-trade-off method, *GHS-TTO* Germany and EHS-VAS Germany represent two national value sets [6, 19]

A patient’s VAS valuation serves as the reference for patient benefit. Performance of the two value sets was analyzed in six steps (Additional file 1: Table S1): comparison of raw values, deviations from the reference, correlation with the reference and with an accepted medical endpoint, comparison of quality-adjusted survival, and identification of factors influencing differences from the reference.

Mean absolute error (MAE) of value sets compared with VAS values reported by patients are investigated over the six measurement points. For correlation between patients’ VAS and value sets, Pearson correlation coefficients ρ are analyzed for absolute valuations as well as differences in valuations over time—the latter are a key indicator of effectiveness trend. To estimate confidence intervals, Bootstrap methods were applied for the correlation coefficients. Correlations were investigated for all patients, for the two study arms of inpatients and outpatients, and for the subgroups of the lower and upper quartiles of patients with regard to VAS valuation reported at admission.

External validity is analyzed using an acknowledged clinical measure of quality of life in cardiac patients: the MacNew, specifically its global score [24]. For all patients, Pearson correlations with the MacNew global score are calculated for patients’ own valuations as well as for the two value sets, again for both absolute valuations reported as well as differences in valuations over time.

Overall treatment effect in terms of quality-adjusted survival is captured by multiplying value by the duration it applies to. In case of the choice-based GHS-Germany, this produces traditional quality-adjusted life years (QALYs). For patients’ VAS and EHS-Germany, quality-adjusted survival is based upon experienced health and thus differs from the ex-ante concept of utility-based QALYs. Analyses are conducted for all patients, for the inpatient and outpatient rehabilitation arms of the clinical study, and for the upper and lower quartiles of patients in terms of quality of life reported. These stratifications are intended to reflect the increasing relevance of analyzing patients by subgroups, which is found especially in patient benefit assessment in German drug regulation [25].

Finally, we explain differences between the two value sets and patients’ own reports by repeated measures regression. Explanatory variables include socioeconomic ones such as age and sex, education, and family status as well as health determinants such as smoking and baseline MacNew global score. All effects are further tested on differences with respect to the time point at which average differences were largest.

## Results

The sample in this study comprised a total of 145 patients with 98 patients in the inpatient arm and 47 patients in the outpatient arm. The overall share of women was 22.8 % with 21.4 % in inpatient care and 25.5 % in outpatient care. Age ranged from 26 to 76 years, averaging 55.6 years with 54.2 years in the inpatient arm and 56.2 years in the outpatient arm.

**Fig. 1:**
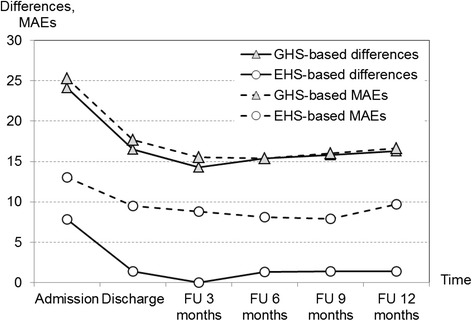
Differences between patients’ reported VAS and values sets, and mean absolute errors (MAEs). Legend: FU, follow-up period after rehabilitation. Differences include respondents with EQ-5D descriptive system not fully completed but VAS reported, number of patients, see Table 1. MAEs include complete responses only, number of patients by observation point: 139, 135, 131, 133, 126, 132

All valuations increased during the rehabilitation phase and remained quite stable during the follow-up period (Table 2). Compared with patients’ direct valuations, TTO-based valuation was on average 24 points higher at admission and then ranged between 14 and 17 points higher during FU (Fig. 1). The EHS-based value set showed an almost eight point higher value at admission but, with a maximum of 1.4, was very close to the reference valuation for the remaining measurements. Both population-based value sets also showed smaller variation than patients’ direct valuation over all measurement points, with the GHS-based value set about a quarter smaller and the EHS-based one about a fifth. MAEs were found to closely match differences for the GHS-based value set whereas, compared with the latter, they were about halved for the EHS-based value set.

**Table 2:** Valuations by patients’ VAS and value sets, six observation points

		Patients’ VAS		GHS-TTO		EHS-VAS	
	Patients	Mean	STD	Mean	STD	Mean	STD
Admission	145	65.1	19.0	89.1	16.9	72.9	15.8
Discharge	138	76.9	16.7	93.4	11.3	78.3	13.4
FU 3 months	135	77.4	15.8	91.7	14.5	77.5	13.8
FU 6 months	136	77.3	16.7	92.7	13.1	78.6	13.6
FU 9 months	128	79.1	15.0	94.9	9.3	80.5	11.4
FU 12 months	133	77.6	16.9	93.9	10.3	79.0	12.6

Legend: *FU* follow-up period after rehabilitation, *STD* standard deviation

**Fig. 2:**
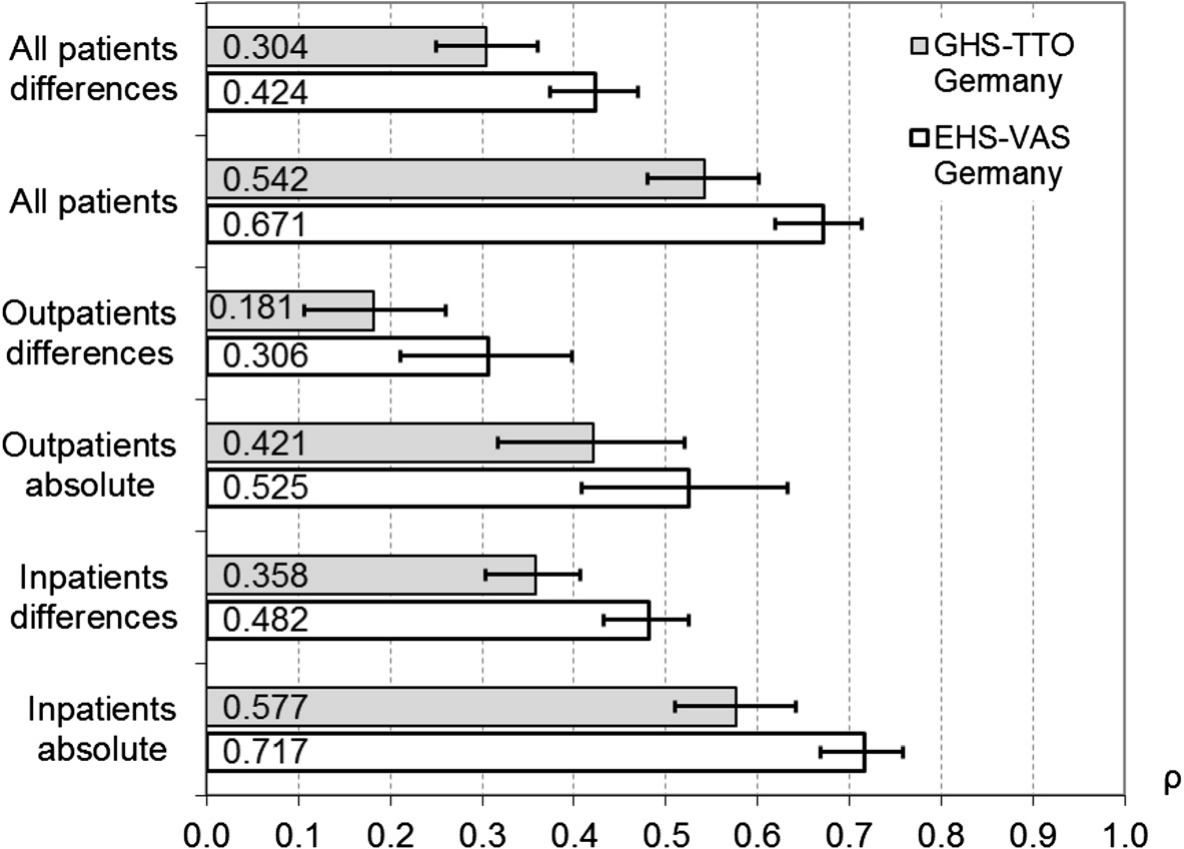
Pearson correlation coefficients (ρ) with patients’ absolute VAS valuations and differences over time. Legend: Two value sets, all observation points; inpatient rehabilitation *n* = 98, outpatient rehabilitation *n* = 47 at admission, otherwise varying number of patients; confidence intervals shown as error bars

Pearson correlation with patients’ VAS was significantly higher for the EHS-based value set than for the GHS-based value set for all patients and for the inpatient treatment arm; it was higher but with overlapping confidence intervals for the outpatient treatment arm (Fig. 2). This structure of results is found for both absolute values as well as differences in values between successive measurements. In the lower quartile of patients with regard to quality of life, correlation with reference values was significantly higher for the EHS-based value set compared with the GHS-based one, for both values and differences in valuations over time. For the upper quarter of patients, correlations were just higher but not significant (Table 3).

**Table 3:** Pearson correlation of value sets with patients’ VAS valuations and their differences

	Lower quartile, patients’ VAS at admission		Upper quartile, patients’ VAS at admission	
	Absolute values	Differences	Absolute values	Differences
GHS-TTO Germany	0.460 (0.333–0.587)	0.233 (0.149–0.320)	0.285 (0.179–0.458)	0.188 (0.112–0.265)
EHS-VAS Germany	0.699 (0.603–0.788)	0.516 (0.430–0.597)	0.420 (0.258–0.571)	0.215 (0.116–0.316)

Legend: Confidence intervals in brackets

**Fig. 3:**
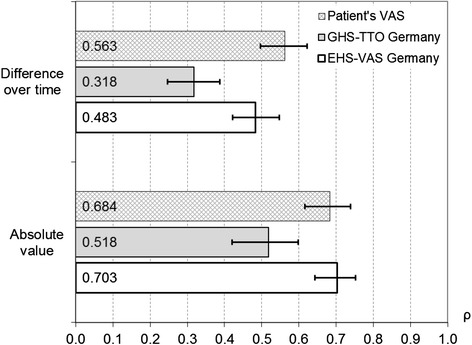
Pearson correlation coefficients of all patients’ absolute VAS valuations with MacNew global score, and their differences over time. Legend: Patients’ VAS and two value sets, all observation points, varying number of patients; confidence intervals shown as error bars

Correlations with MacNew global score were not found to be significantly different for patients’ VAS and the EHS-based value set, for both absolute values and differences in valuations over time; yet both were significantly lower for the GHS-based value set (Fig. 3). For the EHS-based value set, correlation was above 0.7 for absolute values but slightly below 0.5 for differences in valuations over time.

**Fig. 4:**
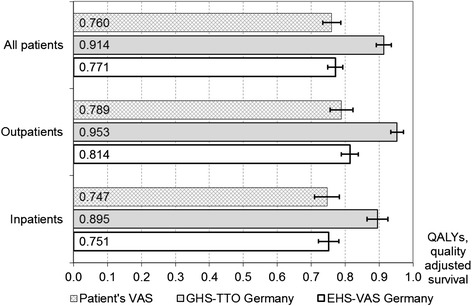
Quality-adjusted survival for patients’ VAS and value sets. Legend: From admission to 12 months follow-up. Indicator reflects QALYs based on ex-ante choices for GHS-TTO Germany and quality-adjusted survival based on experienced health for the other two indicators; confidence intervals shown as error bars

Compared with quality-adjusted survival based on patients’ VAS, only the EHS-based approach did not differ significantly in all patients as well as in the two treatment arms (Fig. 4). However, for all patients (inpatients and outpatients), utility-based QALYs were 20.3, 19.8, and 20.8 % higher than quality-adjusted survival based on patients’ own reports. The add-on effect of using QALYs based on ex-ante choices corresponded to 54 days in perfect health for inpatients and 60 days for outpatients. For quality-adjusted survival calculated from the EHS-based value set, the respective add-on effects were 1 and 9 days.

MAEs between both the EHS-based and the GHS-based value sets and patients’ VAS differed most at “admission”, with an increase of 50 % for the EHS-based value set and 56 % for the GHS-based one. According to the repeated measures regression, these time dependent differences were significantly more pronounced for younger patients. For the EHS-based value set, an additional increase was found for patients with higher level of education and for patients not living alone (Additional file 1: Table S2). For the GHS-based value set, MAEs were larger for patients with lower MacNew values at baseline, over all observation points (Additional file 1: Table S3).

## Discussion

The counterfactual design of this re-analysis enabled comparison with patients’ own valuations that were used as a reference. In most analyses, the population-based estimates of the EHS-based approach were found to closely reproduce the reference of patient-reported valuations. This was especially pronounced for the mean differences between reference and value set, which also reflect potential bias. It also notably existed for mean absolute errors that integrate all individual variation. In addition, differences in correlations for all patients and for subgroups investigated underscored the closer relation of the EHS-based value sets to patient-reported outcome. As the standard used in economic evaluation studies, the GHS-based value set was found to be systematically less strongly related to patients’ reports including both absolute valuations and differences in valuations over time, and also to render systematically higher levels of overall patient benefit in terms of quality-adjusted survival. The latter corresponds to earlier findings that the GHS-based value set tends to underestimate VAS values reported for health states with severe problems, and to overestimate them for states with no or moderate problems [9, 23]. In the present study, the share of patients reporting a severe problem in at least one of the EQ-5D dimensions was 7.6 % at admission, and reduced to 3.0 % at 12 months follow-up. The GHS-based value set describes a larger gain for these patients than they report on the VAS, thus contributing to a higher level of quality-adjusted survival.

EHS-based results were not only more strongly linked to the benefits directly derived from patients, but even showed lower dispersion than these reference values. However, they were found not to reproduce valuation appropriately directly after acute treatment for myocardial infarction: At admission to rehabilitation, patients’ VAS was much lower than the EHS-based approach. Acute live-threatening experience may thus hardly be reflected in the population sample from which the EHS-based value set has been estimated. In particular, younger patients with a higher level of education and not living alone tended to report VAS values lower than the EHS-based value set. The experience of these subgroups was not fully reflected by estimates of population experience.

Patients analyzed had presented with different types of acute cardiac events, although the study design does not allow for extrapolation of findings to all post-acute heart patients. A main methodological limitation of this study is that the two national value sets used are based on quite different concepts: GHS-based valuation is suited to decision makers who intend to allocate money according to ex-ante preferences of the population regarding health. EHS-based valuation aims to derive patient benefit from an average experience of a population sample. It is thus suited to decision makers whose priority is to assess benefit from the patient’s perspective. Decision makers have to make a normative choice about which concept they want to use when appraising the evidence. Given that the EHS-based value set is conceptually more closely related to the reference of patients’ VAS, it could be expected that it may render better estimates for the reference. It is well known that the TTO method tends to produce higher values than VAS valuation, for example when comparing national value sets based on these two methods [1–3]. Quantifying the relative influence of valuation method (VAS, TTO) and type of health state values (GHS, EHS) would have required comparison of four types of value sets which were not available. Yet, for a clinical study on heart rehabilitation patients, three decision relevant points were elaborated here by comparing patient-reported outcome with two value sets: 1. it was shown to what extent traditional, utility-based quality of life measurement and QALYs reflect patient benefit; 2. it was quantified to what extent the normative choice between the two value sets affects effectiveness outcome; and 3. it was shown that the EHS-based value set offers an option to estimate patient-reported outcomes while identifying situations in which estimation is not accurate.

A methodological limitation is that the GHS-based value set has been anchored for the state of being dead, whereas the EHS-based concept could not be anchored for consistency. Aside from methodological discussion about anchoring [26], studies have shown that the impact of anchoring on results may be minor in general population samples [27], which is where the EHS-based approach has been derived from.

Another important point is that the original SARAH study only included patients from the German health care system. The present study thus could not investigate the transfer of quality of life results between health systems. In order to fully quantify the transfer problem, comparison between value sets of identical methodology adapted to two health systems would be needed. Results from an EHS-based value set may yet be used to check whether the outcomes of a clinical study are sensitive to the valuation approach. Decision makers can thus be informed about whether or not valuation methods and transfer problems may play a role in the assessment of patient benefit.

A last conceptual limitation is that, with regard to a specific health care system, only the valuation step has been considered. A similar type of problem might eventually occur for the description of quality of life, although this was not included in the scope of this study.

## Conclusion

To jurisdictions responsible for market access, the concept of valuing quality of life is an issue of salient relevance. For a clinical intervention study, this analysis is, to the best of our knowledge, the first to quantify the impact on outcomes measured of using an EHS-based value set instead of the traditional GHS-based approach. Decision makers who consider patient relevant benefit should especially take into account possible differences between traditional economic utilities and patient-reported outcomes.

The results provide a new option to those who give priority to patient-reported outcomes and to results derived from their target population: In order to adapt quality of life results from clinical studies that have been derived in other populations or have not been fully based on patients’ reports, the EHS-based value set may be used to estimate patients’ valuations. This appropriately achieved, the resulting clinical endpoint may better reflect patient benefit, and may thus bring closer together clinical and economic evaluations.

The performance of the EHS-based estimation is very promising, but the results also indicated that, in situations close to acute vital events, estimates of general population experience may not fully reproduce the patients’ perspective. Yet, the performance of EHS-based value sets in clinical populations different from the one investigated here needs to be tested before use.

### Additional files

An additional file shows tables giving an overview on performance analysis and on repeated measures regression to explain absolute differences between the value set and patients’ VAS [see Additional file 1.doc].

## Additional file

Additional file 1:
**Table S1.** Overview on performance analysis. **Table S2.** Repeated measures regression to explain absolute difference between EHS-based value set and patient’s VAS. **Table S3.** Repeated measures regression to explain absolute difference between GHS-based value set and patient’s VAS. (DOC 77 kb)
