# Supplementary material for: Assessing quality of life in a clinical study on heart rehabilitation patients: how well do value sets based on given or experienced health states reflect patients’ valuations?
Source: Health Qual Life Outcomes. 2016 Mar 22;14:48. doi: 10.1186/s12955-016-0453-3 (PMC4802660; doi:10.1186/s12955-016-0453-3)
Supplement: Additional file 1: — Table S1. Overview on performance analysis. Table S2. Repeated measures regression to explain absolute difference between EHS-based value set and patient’s VAS. Table S3. Repeated measures regression to explain absolute difference between GHS-based value set and patient’s VAS. (DOC 77 kb) [file 12955_2016_453_MOESM1_ESM.doc]

**Additional file 1**

Table S1: Overview on performance analysis

| Criterion to measure performance of value sets | Patient groups analyzed | Time points | Reported in |
| --- | --- | --- | --- |
| Values (Mean, standard deviation) | All patients | All 6 time points | Table 1 |
| Mean absolute error vs. patient reported valuation (absolute and differences from last measurement) | All patients, inpatients, outpatients | All 6 time points | Figure 1 |
| Pearson correlation with patient reported valuation (absolute and differences from last measurement) | All patients, lower and upper quartiles of valuation at admission | Full study period only | Figure 2, Table 3 |
| Pearson correlation with MacNew (absolute and differences from last measurement point) | All patients | Full study period only | Figure 3 |
| Quality adjusted survival/quality adjusted life years | All patients, inpatients, outpatients | Full study period only | Figure 4 |
| Determinants of differences between value set and patient reported valuation (repeated measurement regression) | All patients | Full study period only | Tables S2 and S3 |

Table S2: Repeated measures regression to explain absolute difference between EHS-based value set and patient’s VAS

| Parameter | Estimate | Standard Error | 95% Confidence Limits | | Z | Pr > |Z| |
| --- | --- | --- | --- | --- | --- | --- |
| Intercept | 7.7794 | 0.6546 | 6.4964 | 9.0623 | 11.88 | <.0001 |
| Admission | 2.3810 | 1.9414 | -1.4240 | 6.1860 | 1.23 | 0.2200 |
| Female | -0.5593 | 1.1481 | -2.8096 | 1.6910 | -0.49 | 0.6261 |
| Age^a^ | 0.0390 | 0.0415 | -0.0424 | 0.1204 | 0.94 | 0.3479 |
| Education not low | -1.7383 | 0.9751 | -3.6494 | 0.1728 | -1.78 | 0.0746 |
| Non-smoker | -0.6859 | 0.8961 | -2.4422 | 1.0704 | -0.77 | 0.4440 |
| Living not alone | 2.1176 | 0.8001 | 0.5494 | 3.6859 | 2.65 | **0.0081** |
| Female * admission | 1.2368 | 2.7359 | -4.1255 | 6.5991 | 0.45 | 0.6512 |
| Age ^a^ * admission | -0.1967 | 0.1002 | -0.3931 | -0.0002 | -1.96 | **0.0498** |
| Admission * education not low | 5.1050 | 2.3230 | 0.5519 | 9.6580 | 2.20 | **0.0280** |
| Admission * not living alone | 2.3839 | 2.0328 | -1.6003 | 6.3681 | 1.17 | 0.2409 |
| Admission * non-smoker | -2.3096 | 2.0170 | -6.2629 | 1.6437 | -1.15 | 0.2522 |
| MacNew ^b^ | -0.6824 | 0.4098 | -1.4856 | 0.1208 | -1.67 | 0.0959 |
| MacNew ^b^ * admission | -0.4611 | 0.9355 | -2.2947 | 1.3726 | -0.49 | 0.6221 |

Legend: ^a^ centered values used, ^b^ MacNew global score used; probabilities < 0.05 in bold.

Table S3: Repeated measures regression to explain absolute difference between GHS-based value set and patient’s VAS

| Parameter | Estimate | Standard Error | 95% Confidence Limits | | Z | Pr > |Z| |
| --- | --- | --- | --- | --- | --- | --- |
| Intercept | 16.2331 | 1.5320 | 13.2304 | 19.2357 | 10.60 | <.0001 |
| Admission | 3.3709 | 2.9091 | -2.3307 | 9.0725 | 1.16 | 0.2466 |
| Female | -1.2933 | 1.9699 | -5.1542 | 2.5676 | -0.66 | 0.5115 |
| Age^a^ | 0.0947 | 0.0756 | -0.0534 | 0.2428 | 1.25 | 0.2102 |
| Education not low | -3.7748 | 1.9806 | -7.6568 | 0.1071 | -1.91 | 0.0567 |
| Non-smoker | -0.0777 | 1.8574 | -3.7181 | 3.5627 | -0.04 | 0.9666 |
| Living not alone | 1.1541 | 1.7818 | -2.3381 | 4.6463 | 0.65 | 0.5172 |
| Female * admission | -0.0179 | 4.2381 | -8.3245 | 8.2887 | 0.00 | 0.9966 |
| Age ^a^ * admission | -0.3235 | 0.1402 | -0.5984 | -0.0487 | -2.31 | **0.0210** |
| Admission * education not low | 7.0859 | 4.1578 | -1.0633 | 15.2352 | 1.70 | 0.0883 |
| Admission * not living alone | 6.3159 | 3.4373 | -0.4211 | 13.0529 | 1.84 | 0.0661 |
| Admission * non-smoker | -1.6001 | 3.2983 | -8.0646 | 4.8645 | -0.49 | 0.6276 |
| MacNew ^b^ | -2.3237 | 0.7323 | -3.7589 | -0.8884 | -3.17 | **0.0015** |
| MacNew ^b^ * admission | -0.7781 | 1.4517 | -3.6234 | 2.0671 | -0.54 | 0.5919 |

Legend: ^a^ centered values used, ^b^ MacNew global score used; probabilities < 0.05 in bold.
